# Phylogenetic convolutional neural networks in metagenomics

**DOI:** 10.1186/s12859-018-2033-5

**Published:** 2018-03-08

**Authors:** Diego Fioravanti, Ylenia Giarratano, Valerio Maggio, Claudio Agostinelli, Marco Chierici, Giuseppe Jurman, Cesare Furlanello

**Affiliations:** 1 Fondazione Bruno Kessler (FBK), Via Sommarive 18 Povo, Trento, I-38123 Italy; 2 Max Planck Institute for Intelligent Systems, Spemannstraße 34, Tübingen, 72076 Germany; 3 Centre for Medical Informatics, Usher Institute, University of Edinburgh, 9 Little France Road, Edinburgh, EH16 4UX UK; 4 Department of Mathematics, University of Trento, Via Sommarive 14 Povo, Trento, I-38123 Italy

**Keywords:** Metagenomics, Deep learning, Convolutional neural networks, Phylogenetic trees

## Abstract

**Background:**

Convolutional Neural Networks can be effectively used only when data are endowed with an intrinsic concept of neighbourhood in the input space, as is the case of pixels in images. We introduce here Ph-CNN, a novel deep learning architecture for the classification of metagenomics data based on the Convolutional Neural Networks, with the patristic distance defined on the phylogenetic tree being used as the proximity measure. The patristic distance between variables is used together with a sparsified version of MultiDimensional Scaling to embed the phylogenetic tree in a Euclidean space.

**Results:**

Ph-CNN is tested with a domain adaptation approach on synthetic data and on a metagenomics collection of gut microbiota of 38 healthy subjects and 222 Inflammatory Bowel Disease patients, divided in 6 subclasses. Classification performance is promising when compared to classical algorithms like Support Vector Machines and Random Forest and a baseline fully connected neural network, e.g. the Multi-Layer Perceptron.

**Conclusion:**

Ph-CNN represents a novel deep learning approach for the classification of metagenomics data. Operatively, the algorithm has been implemented as a custom Keras layer taking care of passing to the following convolutional layer not only the data but also the ranked list of neighbourhood of each sample, thus mimicking the case of image data, transparently to the user.

## Background

Biological data is often complex, heterogeneous and hard to interpret, thus a good testbed for Deep Learning (DL) techniques [1]. The superiority of deep neural network approaches is acknowledged in a first group of biological and clinical tasks, with new results constantly flowing in in the literature [2–4]. However, DL is not yet a “silver bullet” in bioinformatics; indeed a number of issues are still limiting its potential in applications, including limited data availability, result interpretation and hyperparameters tuning [5]. In particular, DL approaches has so far failed in showing an advantage in metagenomics, either in terms of achieving better performance or detecting meaningful biomarkers. This lack of significant results led Ditzler and coauthors [6] to state that deep learning “may not be suitable for metagenomic application”; nevertheless, novel promising attempts have recently appeared [7, 8]. With a slight abuse of notation, in what follows we use the more common term metagenomics even in the 16S metabarcoding case, following the notation of the MetaHIT paper [9] and the official Illumina documentation [10].

Unique among other omics, metagenomics features are endowed with a hierarchical structure provided by the phylogenetic tree defining the bacterial clades. In detail, samples are usually described by features called Operational Taxonomic Units (OTU). For each OTU, its position as a leaf of the phylogenetic tree and its abundance value in the sample are automatically extracted by bioinformatics analysis. In this work we exploit this hierarchical structure as an additional information for the learning machine to better support the profiling process: this has been proposed before in [11, 12], but only in shallow learning contexts, to support classification or for feature selection purposes. We aim to exploit the phylogenetic structure to enable adopting the Convolutional Neural Network (CNN) DL architecture otherwise not useful for omics data: we name this novel solution *Ph-CNN*. Indeed CNNs are the elective DL method for image classification [13, 14] and they work by convolving subsets of the input image with different filters. The operation is based on the matricial structure of a digital image and, in particular, the concept of neighbours of a given pixel. Using the same architecture for non-image data requires the availability of an analogous proximity measure between features.

In the metagenomics case, such measure can be inherited by the tree structure connecting the OTUs and the neighbourhood are naturally defined once an approprieate tree distance between two OTUs is defined. In this paper, we adopt the patristic distance, i.e., the sum of the lengths of all branches connecting two OTUs on the phylogenetic tree [15]. By definition, the output of a CNN consists of linear combinations of the original input features: this implies that, if Ph-CNN includes more CNN layers, the problem of finding the neighbours of a OTU is shifted into the hardest task of finding the neighbours of a linear combination of OTUs. The workaround here is mapping OTUs into points of a *k*-dimensional metric space preserving distances as well as possible via a MultiDimensional Scaling (MDS) projection [16]: the use of MDS is allowed because the patristic distance is Euclidean [17]. A further refinement is provided by sparsifying MDS via regularized low rank matrix approximation [18] through the addition of the smoothly clipped absolute deviation penalty [19], tuned by cross-validation. A caveat: different topologies of the phyogenetic tree lead to different distance matrices. As pointed out in [20], different softwares can produce very different topologies, thus the choice of the software and its version in the whole metagenomic pipeline play a critical role here as a relevant source of variability, and this is true for all the steps throughout the whole preprocessing workflow.

The convolutional layer combined with the neighbours detection algorithm is operatively implemented as a novel Keras layer [21] called Phylo-Conv. Ph-CNN consists of a stack of Phylo-Conv layers first flattened then terminating with a Fully Connected (Dense) and a final classification layer. The experimental setup is realized as a 10x5-fold cross-validation schema with a feature selection and ranking procedure, implementing the Data Analysis Protocol (DAP) developed within the US-FDA led initiatives MAQC/SEQC [22, 23], to control for selection bias and other overfitting effects and warranting honest performance estimates on external validation data subsets. Top ranking features are recursively selected as the *k*-best at each round, and finally aggregated via Borda algorithm [24]. Model performance is computed for increasing number of best ranking features by Matthews Correlation Coefficient (MCC), the measure that better convey in an unique value the confusion matrix of a classification task, even in the multiclass case [25–27]. Experiments with randomized features and labels are also performed as model sanity check.

We demonstrate Ph-CNN characteristics with experiments on both synthetic and real omics data. For the latter type, we consider Sokol’s lab data [28] of microbiome information for 38 healthy subjects (HS) and 222 inflammatory bowel disease (IBD) patients. The bacterial composition was analysed using 16S sequencing and a total number of 306 different OTUs was found. IBD is a complex disease arising as a result of the interaction of environmental and genetic factors inducing immunological responses and inflammation in the intestine and primarily including ulcerative colitis (UC) and Crohn’s disease (CD). Both disease classes are characterized by two conditions: flare (f), when symptoms reappear or worsen, and remission (r), when symptoms are reduced or disappear. Finally, since CD can affect different parts of the intestine, we distinguish ileal Crohn’s disease (iCD) and colon Crohn’s disease (cCD). Note however that the number of non zero features varies for the different tasks, (defined by disease, condition site) since some features may vanish on all samples of a class.

Synthetic data are constructed mimicking the structure of the IBD dataset. They are generated as compositional data from multivariate normal distributions with given covariances and means: in particular, to provide different complexity levels in the classification task, four different instances of data are generated with different ratios between class means. On both data types, the Ph-CNN architecture than compared with state-of-art shallow algorithms as Support Vector Machines (SVMs) and Random Forest (RF), and with alternative neural networks methods such as Multi-Layer Perceptron (MLPNN).

Moreover, the bacterial genera detected as top discriminating features are consistent with the key players known in the literature to play a major role during the IBD progression. Since the direct use of Ph-CNN on the IBD dataset leads to overfitting after few epochs due to the small sample size, the IBD dataset is used in a transfer learning (domain adaptation) task.

Finally, although described and demonstrated on bacterial metagenomics, Ph-CCN can be applied to every metagenomics datasets whose features are associated to a taxonomy and thus to a tree structure, as in the case of metagenomics of relatively large eukaryotes now appearing in the literature [29].

A preliminary version of the method has been presented as the M.Sc. thesis [30].

## Methods

### Ph-CNN

**Fig. 1:**
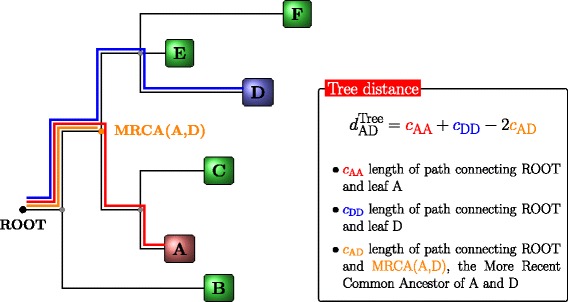
Patristic distance on a tree

The Ph-CNN is a novel DL architecture aimed at effectively including the phylogenetic structure of metagenomics data into the learning process. In detail, Ph-CNN takes as input both the OTU abundances table and the OTU distance matrix described hereafter and provides as output the class of each sample. The core of the network is the Phylo-Conv layer, a novel Keras [21] layer coupling convolution with the neighbours detection. In a generic Phylo-Conv layer, the structure input is represented by a collection of meta-leaves, i.e. linear combinations of the leaves of the original tree; for the first Phylo-Conv layer, the structure input is simply the original set of leaves (OTUs, in the metagenomic case). The neighbour detection procedure identifies the *k*-nearest neighbours of a given metaleaf: the linear combination of the abundances of the corresponding OTUs is then convolved with the filters by the CNN. The core ingredient is the choice of a metric on the phylogenetic tree [31, 32] quantifying the distance between two leaves on the tree. In the current case, we choose the patristic distance [15], i.e., the sum of the lengths of all branches connecting two OTUs. In Fig. 1 we show how to compute the patristic distance between two leaves in a tree. To deal with the problem of finding neighbours for linear combinations of leaves, we map the discrete space of the set of leaves into an Euclidean space of a priori chosen dimension, by associating each leaf to a point *P*_*i*_ in the Euclidean space with variable Euclidean coordinates preserving the tree distance as well as possible. The algorithm used for this mapping is the metric Multidimensional Scaling (MDS) [16], whose use is allowed because the square root $\sqrt {d^{\text {Tree}}}$ of the patristic distance in Fig. 1 is euclidean [17], that is, the matrix (*P*_*i*_·*P*_*j*_) is positive semidefinite. Thus, given a linear combination of OTUs, it is possible to compute its *k*-nearest neighbours as the *k*-nearest neighbours of the corresponding linear combination of projected points *P*_*i*_: in all experiments, the number of neighbours *k* is set to 16. The selected neighbours are then convolved with the 16 filters on the CNN. The Phylo-Conv is then repeated; finally, the terminating layers of the Ph-CNN are a MaxPooling, then a Flatten layer and, finally, a Fully Connected with 64 neurons (changed to 128 for the transfer learning experiments) and a 0.25 Dropout. Each convolutional layer has a Scaled Exponential Linear Units (SELU) [33] as the activation fuction and the dense layer in transfer learning experiments uses a sigmoid activation function. Adam [34] is used as optimizer with learning rate 0.0005.

### Experimental setup

**Fig. 2:**
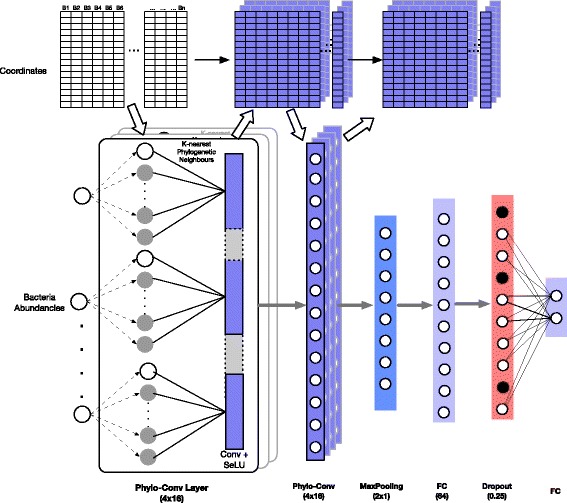
The structure of Ph-CNN. In this configuration, Ph-CNN is composed by two PhyloConv layers followed by a Fully Connected layer before decision

**Fig. 3:**
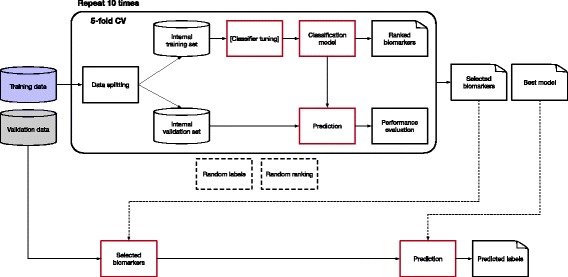
Data Analysis Protocol for the experimental framework

To ensure predictive power and limit overfitting effect, the experimental framework is structured following the guidelines recommended by the US-FDA led studies MAQC/SEQC [22, 23] that investigated the development of predictive models for the analysis of high-throughput data. In particular, the Ph-CNN (shown in Fig. 2) becomes the core of an experimental setup designed according to the DAP shown in Fig. 3, based on 10 repetitions of a 5-fold cross validation. In detail, the dataset is first partitioned into a non overlapping training set and test set, preserving the original stratification, i.e., the ratio between sample size across classes. In the experiments described hereafter, the training set size is 80% of the original dataset. Then the training set undergoes 10 rounds of 5-fold stratified cross validation, with Ph-CNN as the classifier and *k*-Best as the feature selection algorithm, with ANOVA F-value as the ranking score. At each round, several models are built for increasing number of ranked features (in this case, 25%, 50%, 75% and 100% of the total features) using Matthews Correlation Coefficient (MCC) [25, 26] as the performance measure. MCC is rated as an elective choice [22, 23] for effectively combining into a single figure the confusion matrix of a classification task, and hence for evaluating classifiers’ outcomes even when classes are imbalanced. Originally designed for binary discrimination, a multiclass version has also been developed [27, 35]. MCC values range between -1 and 1, where 1 indicates perfect classification, -1 perfect misclassification and 0 for coin tossing or attribution of every samples to the largest class. The lists of ranked features produced within the cross-validation schema are then fused into a single ranked list using the Borda method [36–38]. The subset of the fused list of ranked featured corresponding to the higher MCC value is selected as the optimal set of discriminating features for the classification tasks. The fused list is further used to build the models for increasing number of features on the validation set (sometimes called the external validation set, to avoid ambiguities with the internal validation sets created at each CV round). Finally, as sanity check for the procedure, the same methodology is applied several times on instances of the original dataset after randomly permuting the labels (random labels in Fig. 3) and picking up random features instead of selecting them on the basis of the model performances (random features in Fig. 3): in both cases, a procedure unaffected by systematic bias should return an average MCC close to 0.

### The IBD dataset

The IBD dataset has been originally published in [28] for a study aimed at investigating correlation between bacteria and fungal microbiota in different stages of Inflammatory Bowel Disease. IBD is a clinical umbrella term defining a group of inflammatory conditions of the digestive tract, induced by the interactions of environmental and genetic factors leading to immunological responses and inflammation in the intestine: Ulcerative colitis (UC) and Crohn’s disease (CD) are the two main conditions. The onset of bacterial dysbiosis of the gut microbiota has recently been observed in patients affected by IBD: a decrease in the abundance of *Firmicutes* phylum and an increase for *Proteobacteria* phylum, albeit the exact pathogenesis of IBD remains unknown [39, 40].

The IBD dataset includes both fungal and bacterial abundances from faecal samples of 38 healthy subjects (HS) and 222 IBD patient, collected at the Gastroenterology Department of the Saint Antoine Hospital (Paris, France). In the present study, we only consider the bacterial data subset on which we have a deeper analysis experience.

**Fig. 4:**
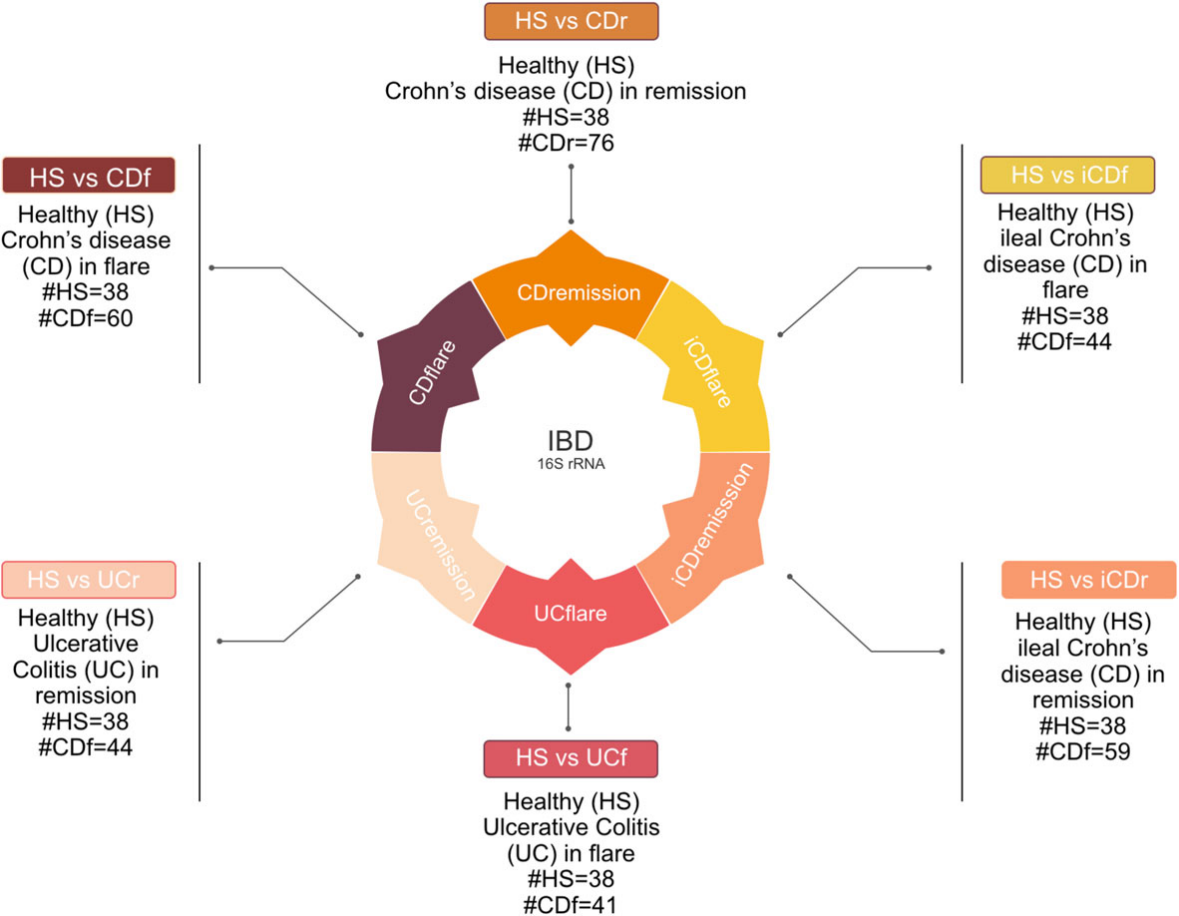
Classification tasks on IDB dataset. The six learning tasks discriminating HS versus different stages of IBD patients

IBD patients are divided in two classes according to the disease phenotype UC and CD. Each disease class is further characterized by two conditions: flare (f), if symptoms reappear or worsen, and remission (r), if symptoms are reduced or disappear. Moreover, since CD can affected different parts of the intestine we further partition the data subset into ileal Crohn’s disease (iCD) and colon Crohn’s disease (cCD). In Table 1 we summarize the sample distribution. In terms of learning tasks, we investigate the six classification tasks discriminating HS versus the six IBD partitions UCf, UCr, CDf, CDr, iCDf and iCDr, as graphically shown in Fig. 4.

**Table 1:** Patient stratification in the IBD dataset

HS	IBD patients					
	CDf		CDr		UCf	UCr
	iCDf	cCDf	iCDr	cCDr		
38	44	16	59	18	41	44
14.6%	16.9%	6.1%	22.7%	6.9%	15.8%	16.9%

**Fig. 5:**
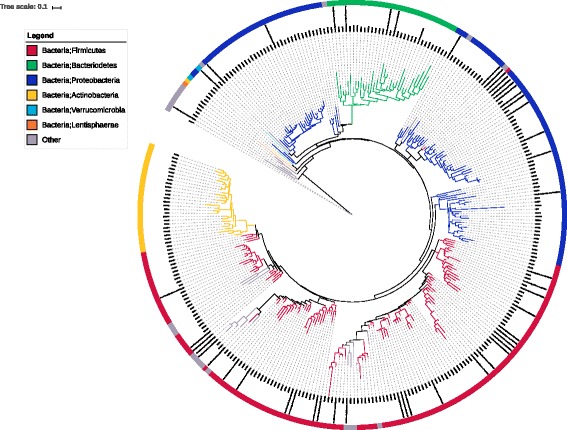
The phylogenetic tree for the IDB dataset

The bacterial composition is analysed using 16S rRNA sequencing, demultiplexed and quality filtered using the QIIME 1.8.0 software [41, 42]; minimal sequence length was 200pb. Sequences are assigned to OTUs using the UCLUST [43] algorithm with 97% threshold pairwise identity and taxonomically classified using Greengenes reference database [44]. Samples with less than 10,000 sequences are excluded from analysis. The number of different OTUs found is 306: each OTU in the data sets is associated to the sequences with the same taxonomy. Among those sequences, the one with the highest median abundance across samples is chosen as the OTU representative. Since many sequences are not in the Greengenes database, OTUs can have an unassigned taxonomy: in this case, the OTU is removed from the analysis. The actual number of OTUs used in the analyses is 259: for some discrimination tasks, however, the number of features is smaller, since some of them are all zeros for all samples in a class. The distance between the OTUs is inferred first by aligning sequences using the NAST algorithm [45, 46] and then by building the phylogenetic tree via the RAxML algorithm [47]. In detail, RaxML has been used in the rapid bootstrap mode with 100 runs, searching for bestscoring Maximum Likelihood tree (best tree). No statistical filter has been applied to the node/edge quality value of the obtained tree. Low supported branches are used as they appear in the RaxML best tree output. The phylogenetic tree for the IBD dataset resulting from the described procedure is shown in Fig. 5: largest abundance values of gut microbiota belong to *Firmicutes* (red), *Bacteroidetes* (green) and *Proteobacteria* (blue), consistently with the published literature. As pointed out already, uncertainties in topology may create fake distances which will ultimately negatively affect all downstream analyses, with software variability playing a major role [20]. While our choice here is to follow the processing pipeline in [28] to ensure data reproducibility, a stronger support in building the phylogenetic tree can be obtained by using alternative algorithms, such as the maximum-likelihood nearest-neighbor interchanges implemented in FastTree2 [48]. Analogous considerations can be formulated for all steps of the preprocessing pipeline: for instance, QIIME is now at version 1.9.1, with major release QIIME 2 scheduled for January 2018. Moreover, the Greengenes database is actually outdated, so switching to another reference database, such as SILVA [49], for the OTU definition would improve the reliability of the process. Finally, the choice to exclude taxonomically unclassified sequences from successive analysis is arbitrary: excluding OTU sequences after a chimera-removal procedure would result in a more precise set of OTUs.

### The synthetic datasets

The synthetic datasets are generated as compositional data, i.e., vectors lying in the *p*-dim Aitchison simplex $\mathcal {S}=\left \{\mathbf {x}=\left (x_{1}, x_{2}, \dotsc, x_{p} \right) \in (\mathbb {R}_{0}^{+})^{p}\;\text {with} ;\sum _{j=1}^{p} x_{j} = 1\right \}$, whose structure resembles the IBD data.

Note that the application of standard multivariate statistical procedures on compositional data requires adopting adequate invertible transformation procedures to preserve the constant sum constrain [50]: a standard map is the isometric log ratio *ilr* [51], projecting the *p*-dimensional Aitchison simplex isometrically to a *p*−1-dimensional euclidian vector. Transformations like *ilr* allow using unconstrained statistics on the transformed data, with inferences mapped back to original compositional data through the inverse map.

The construction of the synthetic data starts from the IDB dataset, and in particular from the two subsets of the HS and CDf samples (by abuse of notation, we use the same identifier for both the class and the compositional data subset). Classes HS and CDf are defined by 259 features (OTU), and they include 38 and 60 samples respectively. The key step is the generation of the synthetic $\text {HS}^{\alpha }_{s}$ and $\text {CDf}^{\alpha }_{s}$ subsets, sampled from multivariate normal distributions with given covariances and mean.

**Fig. 6:**
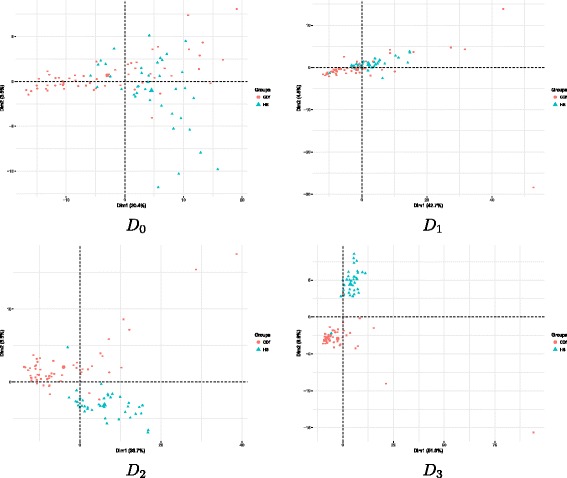
Principal component analysis for the 4 synthetic datasets *D*_0_,*D*_1_,*D*_2_,*D*_3_, with same sample sizes as in the IBD dataset. Larger values of *α* correspond to more separate classes HR and CDf

Operatively, let HS^′^ and CDf^′^ the *ilr*-transformed HS and CDf subsets. Then compute the featurewise mean *μ*(HS^′^)=(*μ*_1_(HS^′^),*μ*_2_(HS^′^),…,*μ*_258_(HS^′^)) and *Σ*(HS^′^) the covariance matrix. Analogously compute *μ*(CDf^′^) and *Σ*(CDf^′^). Consider now the matrix HS0′ defined by substracting to each row of HS^′^ the vector of the means: (HS0′)_*i*·_=(HS^′^)_*i*·_−*μ*(HS^′^), and define analogousy the matrix CDf0′ by (CDf0′)_*i*·_=(CDf^′^)_*i*·_−*μ*(HS^′^). Introduce the projections $P_{\text {HS}'}=\text {HS}'_{0}\cdot (\mu (\text {HS}')-\mu (\text {CDf}'))\phantom {\dot {i}\!}$ and $\phantom {\dot {i}\!}P_{\text {CDf}'}=\text {CDf}'_{0}\cdot (\mu (\text {HS}')-\mu (\text {CDf}'))$, then define now $\sigma =\sqrt {\frac {\sum _{i=1}^{38} (P_{\text {HS}'})_{i}^{2} + \sum _{i=1}^{60} ((P_{\text {CDf}})_{i} - (\mu _{i}(\text {CDf}')-\mu _{i}(\text {HS}')))^{2}} {38+60}}$ and $\mu = \frac {\mu (\text {HS}')+\mu (\text {CDf}')}{2}$. Fix $\alpha \in \mathbb {R}_{0}^{+}$ and define $m_{\text {HS}}=\mu +\alpha \sigma \frac {\mu (\text {HS}')}{||\mu (\text {HS}')||}$ and $m_{\text {CDf}}=\mu +\alpha \sigma \frac {\mu (\text {CDf}')}{||\mu (\text {CDf}')||}$. Then, define $\mathrm {HS'}^{\alpha }_{s}$ as the dataset collecting *n*_HS_ instances from a multivariate normal distribution with mean *m*_HS_ and covariance *Σ*(HS^′^) and analogously $\mathrm {CDf'}^{\alpha }_{s}$. The two synthetic data subsets $\text {HS}^{\alpha }_{s}$ and $\text {CDf}^{\alpha }_{s}$ are defined by taking *ilr*-counterimages: $\text {HS}^{\alpha }_{s} = {ilr}^{-1}\left (\mathrm {HS'}^{\alpha }_{s}\right)$ and $\text {CDf}^{\alpha }_{s} =  {ilr}^{-1}\left (\mathrm {CDf'}^{\alpha }_{s}\right)$. Finally, the synthetic dataset *D*_*α*_ is then obtained as the union $\text {HS}^{\alpha }_{s}\cup \text {CDf}^{\alpha }_{s}$. Setting the parameter *α*, we provide different complexity levels in the classification task. For instance, for *α*=0 the means of the two classes in the synthetic dataset *D*_0_ are the same, while for *α*=1 the means of the two classes HS and CDf are the same as in the IBD dataset; larger values of *α* correspond to easier classification tasks. Principal component analysis of the four datasets *D*_0_,*D*_1_,*D*_2_,*D*_3_ with same sample size as IBD dataset is displayed in Fig. 6.

With the same procedure, a synthetic dataset **D** is created with 10,000 samples and *α*=1, preserving class size ratios.

In practice, generation of the synthetic datasets was performed using the R packages *compositions* [52] and *mvtnorm* [53].

## Results and discussion

The 10×5−fold CV DAP has been applied on instances of the synthetic datasets and on the IBD datasets, comparing the performance with standard (and shallow) learning algorithms such as linear Support Vector Machines (SVM) and Random Forest (RF), and with a standard Multi Layer Perceptron (MLPNN) [54]. As expected [55], no classification task can be reliably tackled by Ph-CNN using the IBD dataset alone: the very small sample size causes the neural network to overfit after just a couple of epochs. To overcome this issue we explore the potentialities of transfer learning.

As a first experiment, we apply the DAP on **D**. In this case, the SELU activation function is used for every layer. The results of the Ph-CNN DAP on **D** are listed in Tables 2, 3, 4, 5, 6, 7 (internal validation) and Table 8 (external validation) on the six classification tasks Healthy vs. {UCf, UCr, CDf, CDr, iCDf and iCDr}; MCC on DAP internal validation is shown with 95% studentized bootstrap confidence intervals [56].

**Table 2:** Dataset **D**: classification performance of Ph-CNN compared to other classifiers on Healthy vs. UCf classification task

UCf	Ph-CNN			LSVM		
*p*	MCC	min CI	max CI	MCC	min CI	max CI
63	0.794	0.785	0.803	0.799	0.793	0.803
125	0.852	0.845	0.860	0.861	0.857	0.865
188	0.920	0.916	0.925	0.924	0.921	0.926
250	0.940	0.937	0.944	0.943	0.941	0.945
	MLPNN			RF		
*p*	MCC	min CI	max CI	MCC	min CI	max CI
63	0.701	0.692	0.721	0.729	0.723	0.736
125	0.838	0.834	0.842	0.843	0.837	0.849
188	0.865	0.861	0.869	0.902	0.899	0.906
250	0.898	0.894	0.901	0.903	0.900	0.907

The performance measure is MCC, with 95% studentized bootstrap confidence intervals (min CI, max CI). Models are computed for *p*={25*%*,50*%*,75*%* and 100*%*} of total number of features for each task. Comparing algorithms are linear Support Vector Machines (LSVM), Random Forest (RF) and MultiLayer Perceptron (MLPNN)

**Table 3:** Dataset **D**: classification performance of Ph-CNN compared to other classifiers on Healthy vs. UCr classification task

UCr	Ph-CNN			LSVM		
*p*	MCC	min CI	max CI	MCC	min CI	max CI
60	0.861	0.855	0.867	0.811	0.807	0.815
119	0.893	0.888	0.899	0.866	0.862	0.870
178	0.906	0.900	0.911	0.892	0.888	0.895
237	0.920	0.916	0.924	0.917	0.914	0.920
	MLPNN			RF		
*p*	MCC	min CI	max CI	MCC	min CI	max CI
60	0.873	0.869	0.443	0.797	0.792	0.801
119	0.877	0.873	0.877	0.799	0.794	0.803
178	0.859	0.855	0.880	0.791	0.787	0.794
237	0.849	0.844	0.854	0.790	0.786	0.795

The performance measure is MCC, with 95% studentized bootstrap confidence intervals (min CI, max CI). Models are computed for *p*={25*%*,50*%*,75*%* and 100*%*} of total number of features for each task. Comparing algorithms are linear Support Vector Machines (LSVM), Random Forest (RF) and MultiLayer Perceptron (MLPNN)

**Table 4:** Dataset **D**: classification performance of Ph-CNN compared to other classifiers on Healthy vs. CDf classification task

CDf	Ph-CNN			LSVM		
*p*	MCC	min CI	max CI	MCC	min CI	max CI
65	0.785	0.775	0.795	0.781	0.776	0.785
130	0.832	0.825	0.840	0.833	0.829	0.838
195	0.896	0.891	0.901	0.910	0.907	0.912
259	0.927	0.924	0.930	0.920	0.918	0.923
	MLPNN			RF		
*p*	MCC	min CI	max CI	MCC	min CI	max CI
65	0.604	0.593	0.614	0.764	0.760	0.769
130	0.821	0.817	0.825	0.805	0.800	0.810
195	0.830	0.825	0.836	0.863	0.860	0.867
259	0.858	0.854	0.862	0.880	0.877	0.883

The performance measure is MCC, with 95% studentized bootstrap confidence intervals (min CI, max CI). Models are computed for *p*={25*%*,50*%*,75*%* and 100*%*} of total number of features for each task. Comparing algorithms are linear Support Vector Machines (LSVM), Random Forest (RF) and MultiLayer Perceptron (MLPNN)

**Table 5:** Dataset **D**: classification performance of Ph-CNN compared to other classifiers on Healthy vs. CDr classification task

CDr	Ph-CNN			LSVM		
*p*	MCC	min CI	max CI	MCC	min CI	max CI
65	0.714	0.705	0.723	0.740	0.734	0.746
129	0.799	0.793	0.806	0.802	0.798	0.808
193	0.850	0.844	0.856	0.860	0.857	0.864
257	0.890	0.884	0.895	0.880	0.877	0.882
	MLPNN			RF		
*p*	MCC	min CI	max CI	MCC	min CI	max CI
65	0.498	0.473	0.521	0.688	0.682	0.695
129	0.783	0.778	0.788	0.744	0.740	0.784
193	0.766	0.759	0.773	0.762	0.756	0.767
257	0.788	0.782	0.794	0.765	0.761	0.771

The performance measure is MCC, with 95% studentized bootstrap confidence intervals (min CI, max CI). Models are computed for *p*={25*%*,50*%*,75*%* and 100*%*} of total number of features for each task. Comparing algorithms are linear Support Vector Machines (LSVM), Random Forest (RF) and MultiLayer Perceptron (MLPNN)

**Table 6:** Dataset **D**: classification performance of Ph-CNN compared to other classifiers on Healthy vs. iCDf classification task

iCDf	Ph-CNN			LSVM		
*p*	MCC	min CI	max CI	MCC	min CI	max CI
62	0.781	0.772	0.790	0.804	0.799	0.808
124	0.863	0.854	0.871	0.861	0.858	0.865
186	0.922	0.918	0.926	0.921	0.919	0.924
247	0.944	0.941	0.947	0.941	0.939	0.942
	MLPNN			RF		
*p*	MCC	min CI	max CI	MCC	min CI	max CI
62	0.845	0.840	0.849	0.748	0.743	0.753
124	0.889	0.886	0.893	0.808	0.803	0.814
186	0.879	0.875	0.883	0.880	0.877	0.883
247	0.901	0.899	0.904	0.890	0.887	0.893

The performance measure is MCC, with 95% studentized bootstrap confidence intervals (min CI, max CI). Models are computed for *p*={25*%*,50*%*,75*%* and 100*%*} of total number of features for each task. Comparing algorithms are linear Support Vector Machines (LSVM), Random Forest (RF) and MultiLayer Perceptron (MLPNN)

**Table 7:** Dataset **D**: classification performance of Ph-CNN compared to other classifiers on Healthy vs. iCDr classification task

iCDr	Ph-CNN			LSVM		
*p*	MCC	min CI	max CI	MCC	min CI	max CI
65	0.753	0.744	0.763	0.773	0.769	0.779
129	0.830	0.823	0.837	0.834	0.830	0.837
193	0.884	0.878	0.889	0.893	0.891	0.896
257	0.910	0.905	0.915	0.907	0.904	0.909
	MLPNN			RF		
*p*	MCC	min CI	max CI	MCC	min CI	max CI
63	0.807	0.802	0.812	0.724	0.719	0.729
125	0.822	0.816	0.827	0.794	0.788	0.800
188	0.831	0.827	0.835	0.812	0.807	0.818
250	0.837	0.831	0.842	0.820	0.816	0.825

The performance measure is MCC, with 95% studentized bootstrap confidence intervals (min CI, max CI). Models are computed for *p*={25*%*,50*%*,75*%* and 100*%*} of total number of features for each task. Comparing algorithms are linear Support Vector Machines (LSVM), Random Forest (RF) and MultiLayer Perceptron (MLPNN)

**Table 8:** Dataset **D**: classification performance of Ph-CNN compared to other classifiers on the external validation dataset

Task	Ph-CNN	LSVM	MLPNN	RF
UCf	0.946	0.934	0.898	0.869
UCr	0.897	0.904	0.897	0.756
CDf	0.926	0.935	0.884	0.859
CDr	0.888	0.888	0.821	0.722
iCDf	0.931	0.943	0.905	0.863
iCDr	0.901	0.910	0.846	0.778

The second experiment is based on a domain adaptation strategy. The Ph-CNN is first trained on the synthetic dataset **D**, then all layer but the last one are frozen, the last layer is substituted by a 2-neurons Dense layer and then retrained on the IBD dataset. Since only the last layer is trained in the second step, the term domain adaptation is best describing the methodology rather than the more generic transfer learning. Here, the activation function is the ReLU for every layer. The results of the Ph-CNN DAP together with the comparing classifiers are listed in Tables 9, 10, 11, 12, 13, 14 (internal validation) and Table 15 (external validation).

**Table 9:** Dataset **D** on IBD: classification performance of Ph-CNN compared to other classifiers on Healthy vs. UCf classification task

UCf	Ph-CNN			LSVM		
*p*	MCC	min CI	max CI	MCC	min CI	max CI
63	0.659	0.604	0.709	0.510	0.449	0.573
125	0.668	0.595	0.734	0.438	0.368	0.500
188	0.650	0.599	0.707	0.541	0.438	0.604
250	0.628	0.567	0.687	0.565	0.510	0.619
	MLPNN			RF		
*p*	MCC	min CI	max CI	MCC	min CI	max CI
63	0.689	0.629	0.743	0.741	0.698	0.783
125	0.644	0.582	0.703	0.742	0.690	0.792
188	0.570	0.496	0.644	0.735	0.680	0.784
250	0.606	0.547	0.667	0.760	0.707	0.816

The performance measure is MCC, with 95% studentized bootstrap confidence intervals (min CI, max CI). Models are computed for *p*={25*%*,50*%*,75*%* and 100*%*} of total number of features for each task. Comparing algorithms are linear Support Vector Machines (LSVM), Random Forest (RF) and MultiLayer Perceptron (MLPNN)

**Table 10:** Dataset **D** on IBD: classification performance of Ph-CNN compared to other classifiers on Healthy vs. UCr classification task

UCr	Ph-CNN			LSVM		
*p*	MCC	min CI	max CI	MCC	min CI	max CI
60	0.445	0.375	0.517	0.509	0.221	0.384
119	0.464	0.393	0.537	0.533	0.238	0.357
178	0.444	0.372	0.520	0.519	0.328	0.449
237	0.346	0.283	0.536	0.408	0.303	0.420
	MLPNN			RF		
*p*	MCC	min CI	max CI	MCC	min CI	max CI
60	0.415	0.350	0.476	0.508	0.425	0.584
119	0.528	0.463	0.596	0.455	0.387	0.525
178	0.538	0.471	0.610	0.435	0.363	0.504
237	0.489	0.417	0.557	0.400	0.337	0.463

The performance measure is MCC, with 95% studentized bootstrap confidence intervals (min CI, max CI). Models are computed for *p*={25*%*,50*%*,75*%* and 100*%*} of total number of features for each task. Comparing algorithms are linear Support Vector Machines (LSVM), Random Forest (RF) and MultiLayer Perceptron (MLPNN)

**Table 11:** Dataset **D** on IBD: classification performance of Ph-CNN compared to other classifiers on Healthy vs. CDf classification task

CDf	Ph-CNN			LSVM		
*p*	MCC	min CI	max CI	MCC	min CI	max CI
65	0.613	0.555	0.665	0.419	0.363	0.472
130	0.617	0.549	0.601	0.326	0.252	0.394
195	0.630	0.560	0.682	0.647	0.595	0.691
259	0.572	0.501	0.620	0.595	0.545	0.642
	MLPNN			RF		
*p*	MCC	min CI	max CI	MCC	min CI	max CI
65	0.610	0.549	0.666	0.677	0.618	0.728
130	0.620	0.551	0.685	0.706	0.648	0.758
195	0.601	0.534	0.667	0.739	0.685	0.788
259	0.648	0.589	0.703	0.720	0.667	0.768

The performance measure is MCC, with 95% studentized bootstrap confidence intervals (min CI, max CI). Models are computed for *p*={25*%*,50*%*,75*%* and 100*%*} of total number of features for each task. Comparing algorithms are linear Support Vector Machines (LSVM), Random Forest (RF) and MultiLayer Perceptron (MLPNN)

**Table 12:** Dataset **D** on IBD: classification performance of Ph-CNN compared to other classifiers on Healthy vs. CDr classification task

CDr	Ph-CNN			LSVM		
*p*	MCC	min CI	max CI	MCC	min CI	max CI
65	0.241	0.172	0.311	0.138	0.073	0.198
129	0.232	0.167	0.295	0.089	0.028	0.151
193	0.202	0.131	0.273	0.169	0.101	0.236
257	0.218	0.158	0.278	0.178	0.107	0.251
	MLPNN			RF		
*p*	MCC	min CI	max CI	MCC	min CI	max CI
65	0.235	0.	0.306	0.488	0.437	0.541
129	0.275	0.199	0.348	0.432	0.373	0.485
193	0.243	0.172	0.315	0.402	0.341	0.464
257	0.233	0.160	0.305	0.398	0.331	0.464

The performance measure is MCC, with 95% studentized bootstrap confidence intervals (min CI, max CI). Models are computed for *p*={25*%*,50*%*,75*%* and 100*%*} of total number of features for each task. Comparing algorithms are linear Support Vector Machines (LSVM), Random Forest (RF) and MultiLayer Perceptron (MLPNN)

**Table 13:** Dataset **D** on IBD: classification performance of Ph-CNN compared to other classifiers on Healthy vs. iCDf classification task

iCDf	Ph-CNN			LSVM		
*p*	MCC	min CI	max CI	MCC	min CI	max CI
62	0.704	0.655	0.753	0.534	0.484	0.583
124	0.702	0.642	0.760	0.414	0.346	0.482
186	0.680	0.614	0.738	0.662	0.605	0.718
247	0.681	0.614	0.739	0.561	0.507	0.621
	MLPNN			RF		
*p*	MCC	min CI	max CI	MCC	min CI	max CI
62	0.679	0.622	0.739	0.787	0.746	0.831
124	0.690	0.634	0.743	0.811	0.766	0.854
186	0.685	0.630	0.742	0.791	0.741	0.836
247	0.708	0.652	0.764	0.775	0.730	0.820

The performance measure is MCC, with 95% studentized bootstrap confidence intervals (min CI, max CI). Models are computed for *p*={25*%*,50*%*,75*%* and 100*%*} of total number of features for each task. Comparing algorithms are linear Support Vector Machines (LSVM), Random Forest (RF) and MultiLayer Perceptron (MLPNN)

**Table 14:** Dataset **D** on IBD: classification performance of Ph-CNN compared to other classifiers on Healthy vs. iCDr classification task

iCDr	Ph-CNN			LSVM		
*p*	MCC	min CI	max CI	MCC	min CI	max CI
65	0.537	0.480	0.601	0.338	0.277	0.409
129	0.522	0.453	0.595	0.319	0.254	0.385
193	0.556	0.492	0.617	0.377	0.315	0.437
257	0.477	0.411	0.544	0.438	0.378	0.492
	MLPNN			RF		
*p*	MCC	min CI	max CI	MCC	min CI	max CI
63	0.526	0.475	0.581	0.552	0.492	0.612
125	0.558	0.493	0.623	0.563	0.516	0.609
188	0.459	0.388	0.527	0.566	0.516	0.616
250	0.529	0.462	0.598	0.539	0.482	0.596

The performance measure is MCC, with 95% studentized bootstrap confidence intervals (min CI, max CI). Models are computed for *p*={25*%*,50*%*,75*%* and 100*%*} of total number of features for each task. Comparing algorithms are linear Support Vector Machines (LSVM), Random Forest (RF) and MultiLayer Perceptron (MLPNN)

**Table 15:** Dataset **D** on IBD: classification performance of Ph-CNN compared to other classifiers on the external validation dataset

Task	Ph-CNN	LSVM	MLPNN	RF
UCf	0.741	0.740	0.666	0.699
UCr	0.583	0.497	0.608	0.678
CDf	0.858	0.642	0.705	0.707
CDr	0.853	0.654	0.654	0.597
iCDf	0.842	0.418	0.401	0.920
iCDr	0.628	0.414	0.414	0.418

**Fig. 7:**
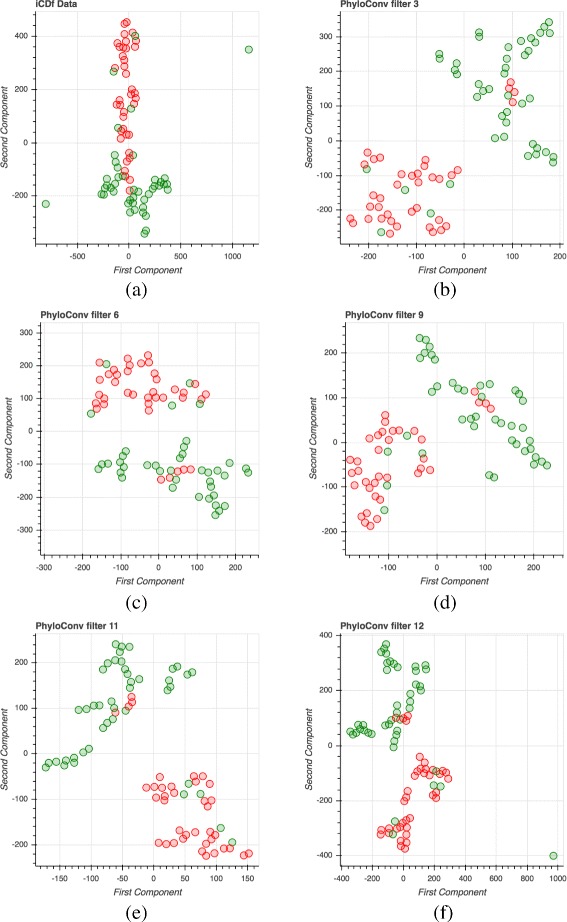
t-SNE projections of the original features at initial layer (subfigure **a**) and after 3, 6, 9, 11, 12 convolutional filters (subfigures **b**-**f**). Green for healthy subjects, red for iCDf patients

As an observation, Ph-CNN tends to misclassify more the samples in class Healthy, rather than those in the other class, for each classification task. In Fig. 7 we show the embeddings of the original features at 6 different levels (after initial input and after 5 PhyloConv filters) for the iCDf task (IBD dataset) by projecting them in two dimensions via t-distributed Stochastic Neighbor Embedding (t-SNE) [57] with perplexity = 5 and 5,000 iterations. While at input level the problem seems hardly separable, the classes tend to form distinct clusters during the flow through convolutional filters applied on OTUs close in the taxonomy.

**Computational details** The Ph-CNN is implemented as a Keras v2.0.8 layer, while the whole DAP is written in Python/Scikit-Learn [58]. All computations were run on a Microsoft Azure platform with 2x NVIDIA Tesla K80 GPUs.

## Conclusions

We introduced here Ph-CNN, a novel DL approach for the classification of metagenomics data exploiting the hierarchical structure of the OTUs inherited by the corresponding phylogenetic tree. In particular, the tree structure is used throughout the prediction phase to define the concept of OTU neighbours, used in the convolution process by the CNN. Results are promising, both in terms of learning performance and biomarkers detection. Extensions of the Ph-CNN architecture are addressing the testing of different tree distances, optimization of neighbours detection and of the number of Phylo-Conv layers. Further, different feature selection algorithms, either generic or DL-specific can be adopted [59–61]. Improvements are expected on the transfer learning and domain adaptation procedures, such as learning on synthetic data and testing on metagenomics, and applying to larger datasets. Finally, beyond the metagenomics applications, we observe that Ph-CNN is a general purpose algorithm, whose use can be extended to other data for which the concept of nearest features can be defined. This is true for all data types that are metrizable, i.e. whenever an embedding exists of the features into a metric space. As an example, we are currently investigating the transcriptomics case, where a grounded distance between genes can be defined by mixing the data-independent Gene Ontology semantic similarity with the correlation between gene expression in the studied dataset [62] through a dedicated multilayer network structure. From a general perspective, the metagenomics and transcriptomics case represent just the first steps towards a more general strategy for effectively exploiting the potential of CNNs, especially for omics data.

## Abbreviations

CD: Crohn’s disease
CNN: Convolutional neural networks
DAP: Data analysis protocol
IBD: Inflammatory bowel disease
MCC: Matthews correlation coefficient MDS: MultiDimensional scaling
MLPNN: Multi-Layer perceptron
OTU: Operational taxonomic unit
RF: Random forest
SVM: Support vector machine
UC: Ulcerative colitis
